# Cytoprotective Effects of Punicalagin on Hydrogen–Peroxide–Mediated Oxidative Stress and Mitochondrial Dysfunction in Retinal Pigment Epithelium Cells

**DOI:** 10.3390/antiox10020192

**Published:** 2021-01-29

**Authors:** Maria Elisabetta Clementi, Giuseppe Maulucci, Giada Bianchetti, Michela Pizzoferrato, Beatrice Sampaolese, Giuseppe Tringali

**Affiliations:** 1 Institute of Chemical Sciences and Technologies “Giulio Natta” (SCITEC)—CNR, L.go F. Vito 1, 00168 Rome, Italy; beatrice.sampaolese@scitec.cnr.it; 2 Biophysics Section, Neuroscience Department, Università Cattolica Del Sacro Cuore, Largo F. Vito 1, 00168 Rome, Italy; giuseppe.maulucci@unicatt.it (G.M.); giada.bianchetti@unicatt.it (G.B.); 3 Fondazione Policlinico Universitario A, Gemelli IRCSS, 00168 Rome, Italy; michela.pizzoferrato@gmail.com; 4 Pharmacology Section, Department of Health Care Surveillance and Bioethics, Università Cattolica del Sacro Cuore, Largo F. Vito 1, 00168 Rome, Italy

**Keywords:** punicalagin, ARPE–19 (human–RPE cell line), mitochondrion, oxidative stress

## Abstract

The retinal pigment epithelium (RPE) is a densely pigmented, monostratified epithelium that provides metabolic and functional support to the outer segments of photoreceptors. Endogenous or exogenous oxidative stimuli determine a switch from physiological to pathological conditions, characterized by an increase of intracellular levels of reactive oxygen species (ROS). Accumulating evidence has elucidated that punicalagin (PUN), the major ellagitannin in pomegranate, is a potent antioxidant in several cell types. The present study aimed to investigate the protective effect of PUN on mitochondrial dysfunction associated with hydrogen peroxide (H_2_O_2_)–induced oxidative stress. For this purpose, we used a human RPE cell line (ARPE–19) exposed to H_2_O_2_ for 24 h. The effects of PUN pre–treatment (24 h) were examined on cell viability, mitochondrial ROS levels, mitochondrial membrane potential, and respiratory chain complexes, then finally on caspase–3 enzymatic activity. The results showed that supplementation with PUN: (a) significantly increased cell viability; (b) kept the mitochondrial membrane potential (ΔΨm) at healthy levels and limited ROS production; (c) preserved the activity of respiratory complexes; (d) reduced caspase–3 activity. In conclusion, due to its activity in helping mitochondrial functions, reducing oxidative stress, and subsequent induction of cellular apoptosis, PUN might be considered a useful nutraceutical agent in the treatment of oxidation–associated disorders of RPE.

## 1. Introduction

The retinal pigment epithelium (RPE) is a dynamic barrier between the retina and the systemic circulation barrier, which is implicated in many fundamental functions that support photoreceptor health and integrity [1,2,3]. The RPE maintains retina and choriocapillaris homeostasis, checking the transport of nutrient substances, removing harmful ones, and releasing neurotrophic and growth factors to and from the retina [4,5]. It is, therefore, not surprising that the integrity, dysfunction, and atrophy of the RPE play important roles in the switch between physiology and pathology [6,7].

Chronic dysfunctions of the RPE are associated with various degenerative ocular pathologies, including age–related macular degeneration or Stargardt disease [8,9]. The aging process and oxidative damage to the RPE, which are linked to high reactive oxygen species (ROS) levels, have been identified as main factors contributing to these pathologies [7,10]. In fact, although the RPE can counteract ROS via different mechanisms, exposure to exogenous stressors may induce an overproduction of ROS and consequently promote an imbalance between production and neutralization, which is the basis of the pathogenesis of degenerative ocular diseases [11,12,13].

Recent scientific evidence suggests that degenerative retinal diseases have a common root: oxidative–stress–induced mitochondrial dysfunction of the RPE cells [14,15]. Due to this, RPE mitochondria have been suggested as new therapeutic targets for these pathologies [16]. In this regard, pre–clinical and clinical studies have highlighted that an adequate supply of antioxidant substances represents a potential therapy for prophylaxis or in treatment of chronic retinal diseases [17,18,19]. In particular, nutraceutical antioxidants have been proven effective as both ROS scavengers and inhibitors or inducers of the mitochondrial signaling pathways related to adaptive responses to oxidative stress [20,21,22,23,24].

Punicalagin (2,3–hexahydroxydiphenoyl–gallagyl–*D*–glucose; PUN), a polyphenol extracted from pomegranate *(Punica granatum*), is a promising multifunctional molecule with anti–inflammatory, antiproliferative, hepatoprotective, and antigenotoxic properties [25,26,27,28,29]. Such properties are mainly ascribed to its antioxidant activity and its ability to promote cellular mitochondrial functions [30,31,32]. In this regard, we previously showed in vitro, and for the first time, that PUN is also able to protect RPE from oxidative damage via the Kelch–like ECH–associated protein–1/nuclear factor erythroid 2–related factor 2/antioxidant responsive elements (Keap/Nrf2/ARE) signaling pathway, confirming its antioxidant properties at the ocular level [33]. However, a better understanding of the mechanisms underlying PUN’s antioxidant activity in the RPE could provide the scientific basis for new therapeutic strategies and options for the prevention or delay of the pathological progression of retinal degenerative diseases. Therefore, the goal of this study was to gain further insight into the potential antioxidant role of PUN in the regulation of mitochondrial function. To this purpose, we used an in vitro H_2_O_2_-induced oxidative damage model based on a human–RPE cell line (h–ARPE 19), which was previously developed and validated by our group [34]. Hence, we examined the relationships among PUN pre–treatment (24 h) and cellular vitality, mitochondrial ROS production, mitochondrial membrane potential (ΔΨm), and activation of caspase–3 in order to determine a possible functional link between the antioxidant effect of PUN supplementation and modulation of mitochondrial function in RPE cells. Moreover, the activity modulation of mitochondrial respiratory complexes by PUN was also investigated. From the present study, it emerges that PUN is an effective and promising mitochondria–targeting antioxidant nutraceutical.

## 2. Materials and Methods

### 2.1. Cell Line and Treatments

ARPE–19 cells (a human RPE cell line) were purchased from the American Type Cell Culture (ATCC–CRL–2302, Manassas, VA, USA). The cells were cultured in basal DMEM/F12 medium (SIGMA D6421; Merck Life Science S.r.l. Milano Italy) supplemented with 10% FBS (Gibco; Thermo Fisher Scientific, Inc., Waltham, MA, USA) and 100 U/mL penicillin–streptomycin in a humidified environment containing 5% CO_2_. Once 80% confluence was achieved, the cells were subcultured and used at a concentration of 50,000 cells/cm^2^ in all experimental conditions, except where required eventually by experimental procedures for the commercial kit.

The cells were pre–treated with punicalagin (PUN) 24 h before inducing damage with hydrogen peroxide (H_2_O_2_). PUN powder (Sigma–Aldrich, St. Louis, MO, USA) was dissolved in ethanol to obtain 10 mM stock solution; further dilutions were made in the incubation medium. All solutions were freshly prepared before each experiment.

### 2.2. Cell Viability

The cell viability was assessed using the MTS assay (Promega srl–Padova–Italy) [35]. In brief, ARPE–19 cells were collected and seeded into 96–well plates at a density of 10,000 cells/well and incubated for 24 h in the presence of increasing concentrations of H_2_O_2_ (range: 25–400 µM). Cell viability was determined by measuring absorbance at 490 nm using a microplate photometer (BioTek™ Elx800-Box 998; BioTek Instruments, Winooski, VT, USA). In the same experimental paradigm, once the optimal cytotoxic concentration was determined, the cells were pre–treated (24 h) with punicalagin at increasing doses (range: 0.5–40 µM) and subsequently treated with 250 µM of H_2_O_2_ for a further 24 h. Results are expressed as the percentage of cell viability relative to the untreated control.

### 2.3. Mitochondria Purification

Mitochondria were isolated from ARPE–19 cells using a Mitochondria/Cytosol Fractionation Kit (MBL, Medical and Biological Laboratories, 200 Dexter Ave., Watertown, MA 02472, USA), according to the supplier’s instructions. Briefly, 50 × 106 pellet cells were incubated at 4 °C for 10 min with 1.0 mL of cytosol extraction buffer mix. Subsequently, the cells were homogenized and centrifuged at 800× *g* for 10 min to remove unbroken cells and nuclei. The supernatant was collected and centrifuged at 15,000× *g* for 10 min at 4 °C. The pellet fraction was solubilized in mitochondrial extraction buffer mix. Protein concentrations were determined using the Thermo Scientific™ Pierce™ BCA Protein Assay Kit (Thermo Fisher Scientific, 3747 N. Meridian Road, Rockford, IL 61101, USA).

### 2.4. Mitochondria ROS Detection

A Mitochondrial ROS Detection Assay Kit (Item # 701600, Cayman Chemical) was used according to the manufacturer’s protocol. The kit is targeted primarily for use in distinguishing mitochondrial ROS from cytoplasmic ones. Briefly, ARPE–19 cells were cultured in a 96–well microplate (25,000 cells per well) and treated at the different experimental conditions. Successively the cells were treated with dihydroethidium (DHE), which is a redox–sensitive fluorescent probe primarily targeting the mitochondrion, and with antimycin A as a positive control for ROS generation. After incubation at 37 °C for 1 h, fluorescence was quantified (excitation wavelength of 480 nm and emission of wavelength 560 nm) using a CytoFluor multi–well plate reader (Victor3–Wallac–1420; PerkinElmer, Waltham, MA, USA). Mitochondrial ROS production was expressed as the fluorescence intensity and presented as a percentage relative to the untreated control.

### 2.5. Confocal Microscopy Imaging and Evaluation of Mitochondrial Membrane Potential

Confocal imaging was performed with a Nikon A1–MP confocal microscope. First, 1 µL of JC–9 1 mM stock solution (Molecular Probes Inc., Eugene, OR, USA) was added per milliliter of culture medium. For the evaluation of mitochondrial membrane potential, images were acquired in two separated channels (excitation: 488 nm, emission: 525/50 nm for the green channel and 595/50 nm for the red channel). The red/green fluorescent ratio, which reflects variations in mitochondrial membrane potential, was calculated as:

(1) $$\frac{R}{G}=\frac{I_{R}}{I_{G}}$$

where $I_{R}$ and $I_{G}$ are the fluorescence emission intensities in the red and green channels, respectively [36,37]. The gain from the detectors and the laser intensity were kept fixed in all the experiments. Mitochondrial depolarization, indicated by a blue shift, corresponds to a decrease in the red/green fluorescent ratio, while a hyperpolarization results in an increased ratio [38].

### 2.6. Mitochondrial Complex I–IV Activity Measurements

The oxidative phosphorylation complex enzymatic activities of mitochondria were measured using specific assay kits according to the manufacturers’ protocols.

Mitochondrial respiratory complex I [Nicotinamide adenine dinucleotide reduced (NADH) oxidase/co–enzyme Q reductase] activity was determined using 20 mg of mitochondrial protein with the MitoCheck Complex I Activity Assay Kit (Cayman Chemicals; catalogue no. 700930 Ann Arbor, MI, USA). Mitochondrial complex I activity was determined by measuring the decrease in NADH oxidation, which is reflected by a decreased in absorbance at 340 nm, in the presence of antimycin A (10 M) to ensure inhibition of complex III.

Activity of mitochondrial complex II (succinate dehydrogenase/co–enzyme Q reductase) was assessed by the rate of reduction of 2,6–dichlorophenolindophenol (DCPIP), which is protonated by reduced co–enzyme Q (Cayman Chemical n° 700940, Ann Arbor, MI, USA) and is reflected by a decrease in absorbance at 600 nm.

Mitochondrial complex III (co–enzyme Q cytochrome c oxidoreductase) activity was determined by the rate of cytochrome c reduction, which is reflected by increased absorbance at 550 nm (Cayman Chemical n° 700950, Ann Arbor, MI, USA). To prevent the backflow of electrons through complex I and the reduction of cytochrome c by complex IV, activity measurements were performed in the presence of 1 mM rotenone and 2 mM sodium azide.

The activity of mitochondrial complex IV (cytochrome c oxidase) was determined by measuring the rate of oxidation of cytochrome c, which is reflected by a decrease in absorbance at 550 nm (Cayman Chemical n° 700990, Ann Arbor, MI, USA).

The activities of respiratory complexes are shown as percentage changes over controls (100%).

### 2.7. Measurement of Caspase–3 Activity

ARPE–19 cells were plated in petri dishes at a seeding density of 3 × 10^6^ cells/35 mm dish; after treatment, cells were harvested by centrifugation. The pellets were washed with PBS, lysed in 50 mL of chilled cell lysis buffer, and left on ice for 10 min. Subsequently, the lysates ware centrifuged at 10,000× *g* for 1 min at 4 °C, and the supernatants were used to assess the caspase–3 assay. The protein concentration in each lysate was measured using a Bicinchoninic Acid (BCA) Protein Assay Kit from Thermo Scientific™ Pierce (Thermo Fisher Scientific | 3747 N. Meridian Road | Rockford, IL 61101, USA).

Caspase–3 activity was determined by using a specific colorimetric assay kit from Sigma Chemical Co. (St. Louis, MO, USA) according to the manufacture’s protocol. DEVD–pNA (Asp–Glu–Val–Asp para–nitroanilide) was used as a colorimetric substrate. The assay was based on spectrophotometric detection of the chromophore p–nitroaniline (pNA) after the caspase–dependent cleavage from the labeled substrate DEVD–pNA using active caspase–3. Caspase–3 activity was determined by quantification of the free pNA using a plate reader at a wavelength of 405 nm. The obtained values were expressed as a percentage relative to the untreated control.

### 2.8. Statistical Analysis

All results are shown as the mean ± SEM (standard error of the mean) of (n) replicates for each experimental group. Each experiment was repeated at least two times in triplicate unless stated otherwise. Significant differences between groups were calculated using one–way analysis of variance (ANOVA) with a subsequent Dunnet or Newman–Keuls post hoc test for comparisons between group means. All data were analyzed using Prism software version 5.1 (GraphPad, Inc., La Jolla, CA, USA). Differences were considered statistically significant at *p* < 0.05.

## 3. Results

### 3.1. Punicalagin Pre-Treatment Attenuates H_2_O_2_–Induced Cell Death and Mitochondrial Oxidative Damage

**Figure 1:**
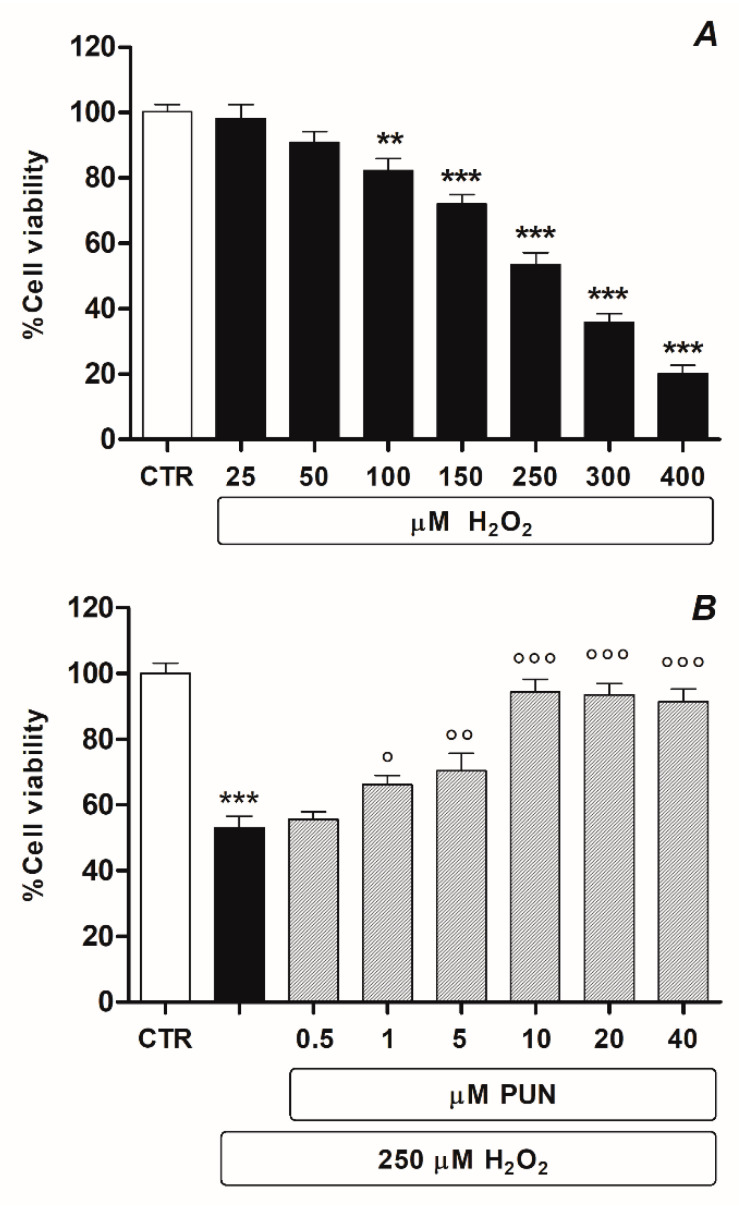
Viability of ARPE–19 cells treated with H_2_O_2_ (range 25–400 µM) for 24 h (**A**) and protective effect of 24 h pre–treatment with multidose PUN (range 0.5–40 µM) (**B**). Data from two independent experiments are expressed as percentage viability values with respect to untreated cells (control = 100%) and are presented as the mean ± SEM of six replicates per experimental group. One–way ANOVA analysis was carried out followed by Dunnett’s or Newman–Keuls test for multiple comparisons with a control or for pairwise comparisons among sample means. Note: ** = *p* < 0.01 and *** = *p* < 0.001 vs. untreated control; ° = *p* < 0.05, °° = *p* < 0.01, °°° = *p* < 0.001 vs. H_2_O_2_ alone.

Human ARPE–19 cell culture was chosen in this study because it is a specific and widely used in vitro experimental model for the study of RPE neurodegenerative diseases. The study’s first set of planned experiments was carried out to investigate the inhibition rate of cell growth by H_2_O_2_ in ARPE–19 cells using an MTS assay. In dose–response experiments, H_2_O_2_ was administered in a dose range of 25 to 400 µM for 24 h. Findings obtained via MTS assay (Figure 1A) highlighted that the rate of cell growth inhibition was directly proportional to the concentration of H_2_O_2_. In particular, the results showed that cell inhibition was approximately 50% (53.5% ± 3.677 n = 6) at a concentration of 250 μM. Therefore, the subsequent experiments were conducted using this concentration to induce cytotoxicity.

Subsequently, experiments were performed in order to evaluate whether or not PUN was capable of reducing H_2_O_2_ cytotoxicity in our experimental model. Dose–response experiments were conducted with PUN in the range of 0.5–40 µM in the presence of 250 µM H_2_O_2_. ARPE–19 cells pre–treated (24 h) with PUN and afterward incubated with H_2_O_2_ (24 h) showed significantly attenuated H_2_O_2_–induced cytotoxicity compared to cells exposed to only H_2_O_2_ alone. The observed protective effect of PUN was concentration–dependent, recovering cell viability of over 90% (94.3% ± 3.88 n = 6) at a concentration of 10 µM (Figure 1B).

In light of the obtained results, the subsequent experiments were conducted with the following experimental protocol: pre–treatment with 10 µM PUN for 24 h followed by the addition of H_2_O_2_ for a further 24 h, except for control groups treated with medium, PUN, or H_2_O_2_ alone.

**Figure 2:**
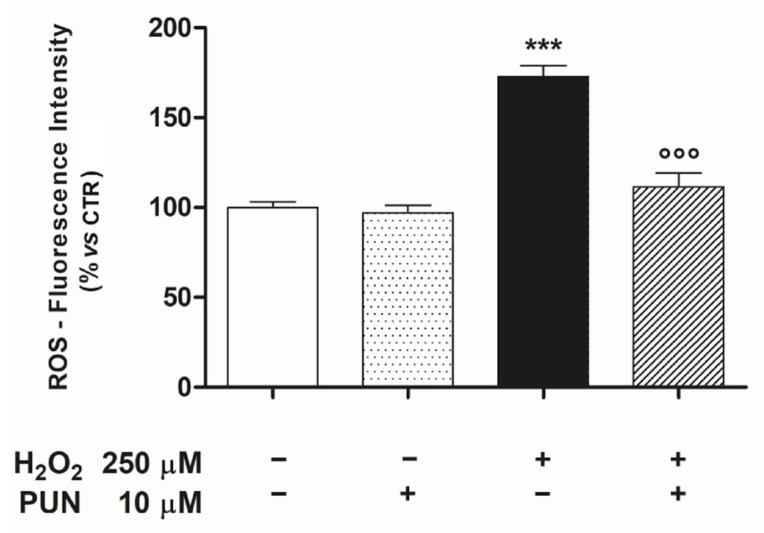
It was possible to reduce the H_2_O_2_–induced increase of mitochondrial ROS levels by pre–treating the ARPE–19 cells with PUN for 24 h. Values are expressed as percentages relative to the untreated control (100%) and are presented as means ± SEM of data from two distinct experiments, each in quadruplicate. One–way ANOVA analysis was carried out followed by post hoc Newman–Keuls test. Note: *** = *p* < 0.001 vs. control; °°° = *p* < 0.001 vs. 250 µM H_2_O_2_ alone.

Next, to unravel if the cytoprotection observed post-PUN treatment was mediated by attenuating mitochondrial ROS, we measured the production of ROS within these organelles in our experimental paradigm. Under these conditions, pre-treatment with PUN was able to reduce H_2_O_2_–induced ROS mitochondrial levels (control: 100.0% ± 3.25, n = 6; PUN: 97.17% ± 4.06, n = 6; H_2_O_2_: 172.67% ± 6.32, n = 6; H_2_O_2_ + PUN 111.93 ± 3.51, n = 6) (Figure 2).

### 3.2. Effect of Punicalagin on H_2_O_2_–Induced Reduction of Mitochondrial Membrane Potential

RPE cells are an energy–intensive cell type and mitochondria are their main source of energy. Since mitochondrial dysfunction is an early event in the H_2_O_2_–induced cell death process, we examined mitochondrial membrane potential in H_2_O_2_–treated ARPE–19 cells, with or without punicalagin pre–treatment, using confocal microscopy imaging.

JC–9 is a ratiometric cationic dye characterized by its potential–dependent accumulation in mitochondria, which is indicated by a shift in the fluorescence emission from green (~525 nm) to red (~590 nm) [39]. This potential–sensitive color is due to the concentration–dependent formation of J–aggregates and variations in the red/green fluorescence intensity ratio, being dependent only on the membrane potential and not on other factors such as the mitochondrial size, shape, and density. It provides an indicator for mitochondrial depolarization, which occurs in the early stage of apoptosis, and allows the responses of cells to an applied stimulus to be assessed [40].

**Figure 3:**
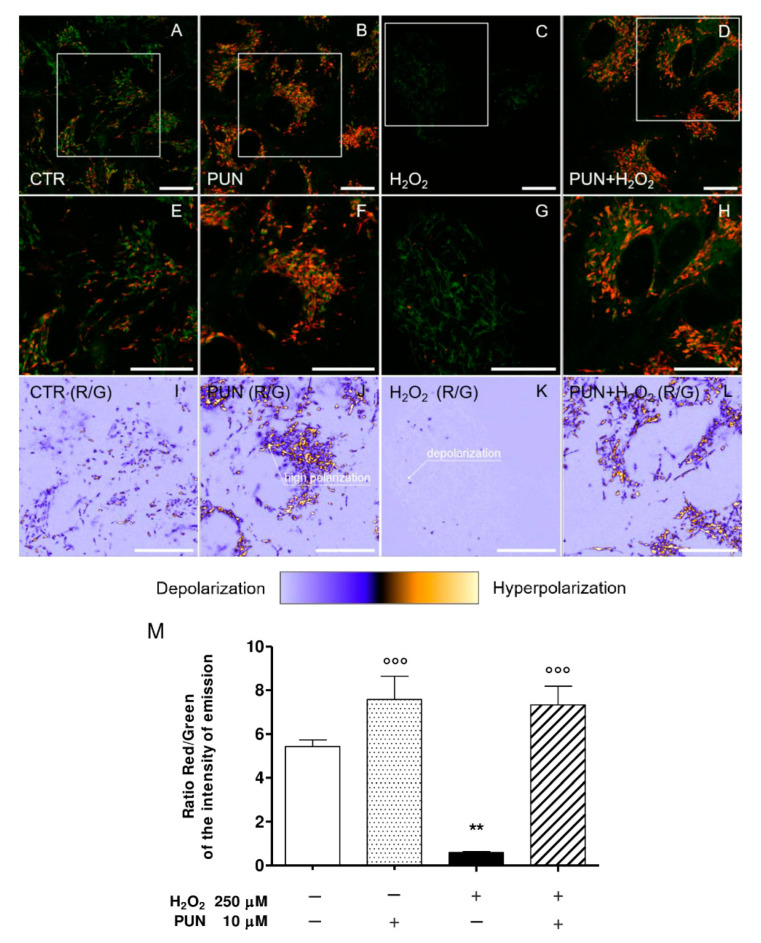
Representative confocal images of ARPE–19 cells untreated (CTR, first column), treated with 10 µM punicalagin (PUN, second column), treated with 250 µM hydrogen peroxide (H_2_O_2_, third column), or pre–treated with punicalagin 24 h before H_2_O_2_ (PUN+ H_2_O_2_, fourth column). Composite dual–channel images (**A**–**D**) are shown in the first row, along with a magnification of CTR (**E**), PUN (**F**), H_2_O_2_ (**G**), and PUN+ H_2_O_2_ (**H**), respectively. The green channel (emission: 525/50 nm) indicates the fluorescence intensity from monomers, while the red channel (emission: 595/50 nm) represents the emissions from aggregates. In the third row, representative maps of the red/green fluorescence intensity ratio (indicated as R/G) are reported for CTR (**I**), PUN (**J**), H_2_O_2_ (**K**), and PUN+ H_2_O_2_ (**L**), respectively. Each pixel’s color spans from light purple (low R/G emission intensity ratio, mitochondrial depolarization) to yellow (high R/G emission intensity ratio, mitochondrial hyperpolarization). Values of the emission intensity ratio range from 0 to 15. The means ± 1 SEM of the ratio are summarized in the bar plot reported in (**M**). Results are from two independent experiments, each including three replicates per experimental group. One–way ANOVA analysis was carried out followed by post hoc Newman–Keuls test. ** = *p* < 0.01 vs. control; °°° = *p* < 0.001 vs. 250 µM H_2_O_2_ alone.

The panel in Figure 3 shows representative confocal images of untreated ARPE–19 cells (CTR; Figure 3A), cells treated with 10 µM punicalagin (PUN; Figure 3B), cells treated with 250 µM hydrogen peroxide (H_2_O_2_; Figure 3C), or cells pre–treated with punicalagin 24 h before H_2_O_2_ (PUN + H_2_O_2_; Figure 3D). In the first row, composite dual–channel images are reported along with a magnification of CTR, PUN, H_2_O_2_, and PUN + H_2_O_2_ images, respectively (Figure 3E–H). The green channel (emission: 525/50 nm) indicates the fluorescence intensity from monomers, while the red channel (emission: 590/50 nm) represents the emissions from J–aggregates. Representative maps of the red/green fluorescence intensity ratio for CTR, PUN, H_2_O_2_, and PUN+H_2_O_2_ cells are shown in Figure 3I–L, respectively, where each pixel is colored according to the red/green intensity ratio, ranging from light purple (low R/G emission intensity ratio, mitochondrial depolarization) to yellow (high R/G emission intensity ratio, mitochondrial hyperpolarization). Mean values ± standard deviations of the ratio are summarized in the bar plot reported alongside with the images (Figure 3M).

At this point of the study, and supported by the results of our previous study [33], we hypothesized that PUN’s effect on the mitochondrial membrane potential, both in terms of the basal conditions and in the presence of H_2_O_2_, is caused by the upstream activation of Keap1–Nrf2 antioxidant defence system. In fact, it is well known that Nrf2 can influence many aspects of mitochondrial physiology, including increasing the mitochondrial membrane potential [41,42].

Treating cells with 250 µM H_2_O_2_ (Figure 3C) induced strong mitochondrial depolarization, as represented in Figure 3 (Figure 3G,K). In particular, comparing the value of the red/green emission intensity ratios for CTR and H_2_O_2_ cells (Figure 3I,K, respectively), a decrease from 5.43 ± 0.54 for the CTR to 0.60 ± 0.08 for H_2_O_2_ was observed. Looking at Figure 3B and the magnified view in Figure 3F, it is possible to observe a higher presence of J–aggregates (red emission) compared to CTR (Figure 3A,E), corresponding to an increase in the R/G emission ratio, as clearly represented in Figure 3I, J, respectively. This suggests a potential protective effect of punicalagin, which can also be achieved in the presence of a subsequent treatment with H_2_O_2_, as shown in Figure 3D,H,L. In this case, although a slight decrease in the R/G ratio from 7.58 ± 1.84 (pre–treatment with punicalagin) to 7.34 ± 1.48 (H_2_O_2_ 24 h after punicalagin pre–treatment) was detected, a significant increase in polarization can be observed with respect to CTR. Overall, these data show that mitochondrial hyperpolarization occurred in both pre–treated samples (PUN and PUN + H_2_O_2_).

### 3.3. Protective Effect of PUN Supplementation on H_2_O_2_–Induced Respiratory Chain Dysfunction in ARPE–19 Cell Mitochondria

A second series of experiments was performed to investigate whether the protective effect of PUN, as previously observed for H_2_O_2_–induced mitochondrial depolarization, was also extended to the mitochondrial respiratory chain. Therefore, enzymatic activities of mitochondrial complexes I (NADH–ubiquinone oxidoreductase), II (Succinate dehydrogenase), III (ubiquinone–cytochrome c oxidoreductase), and IV (cytochrome c oxidase) were assessed in mitochondrial fractions of the ARPE–19 cells in our experimental paradigm.

**Figure 4:**
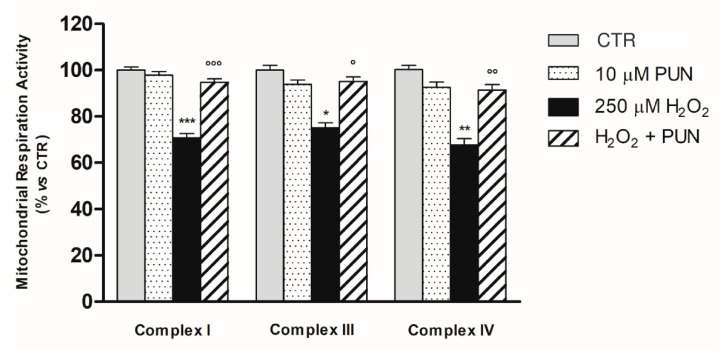
The activities of mitochondrial respiratory chain complexes I, III, and IV were measured in mitochondria isolated of ARPE–19 cells. Enzyme activities were determined using specific assay kits (see Section 2.6 in Material and Methods). Results are shown as percentages relative to control for the single complex, which was set at 100%. Data are presented as the mean ± SEM of six replicates per experimental group from two independent experiments. One–way ANOVA analysis was carried out followed by post hoc Newman–Keuls test for each single respiratory complex. *, *p* < 0.05; ** = *p* < 0.01 and *** = *p* < 0.001 vs. Control; ° = *p* < 0.05; °° = *p* < 0.01 and °°° = *p* < 0.001 vs. H_2_O_2_ (250 µM) alone.

The enzyme activities of mitochondrial complexes I, III, and IV, as displayed in Figure 4, were significantly reduced after exposure to 250 μM H_2_O_2_ for 24 h compared to the control group related to each single complex (*p* < 0.001, *p* < 0.05, and *p* < 0.01, respectively). Interestingly, pre–treatment (for 24 h) with PUN antagonized H_2_O_2_–mediated respiratory chain dysfunction by restoring the enzymatic activity of the respiratory complexes almost to the levels of controls not exposed to oxidative damage (complex I: control: 100.0% ± 3.25; PUN: 97.9% ± 3.53; H_2_O_2_: 70.73% ± 4.52; H_2_O_2_ + PUN 94.79 ± 3.51; complex III: control: 100.0% ± 5.97; PUN: 93.88% ± 4.78; H_2_O_2_: 75.02% ± 5.55; H_2_O_2_ + PUN 95.00 ± 5.01; complex IV: control: 100.0% ± 4.41; PUN: 92.53% ± 5.78; H_2_O_2_: 67.67% ± 6.57; H_2_O_2_ + PUN 91.33 ± 5.99. Six replicates in each experimental group). Conversely, no significant differences between the experimental groups were observed for complex II in our experimental paradigm (control: 100.0% ± 3.24 n = 6; PUN: 97.6% ± 5.31 n = 6; H_2_O_2_: 96.7% ± 5.54 n = 6; H_2_O_2_ + PUN 98.83 ± 4.60 n = 6). Furthermore, it should be noted that contrary to what was seen in the other complexes, the values obtained for complex II were very close to the detection limit of the dosage.

Pre–treatment with punicalagin resulted in no significant changes in mitochondrial respiratory activity in the absence of the oxidative stimulus compared to the control group related to each single complex (Figure 4).

### 3.4. Punicalagin Pre-Treatment Inhibits H_2_O_2_-Induced Caspase–3 Enzymatic Activity

**Figure 5:**
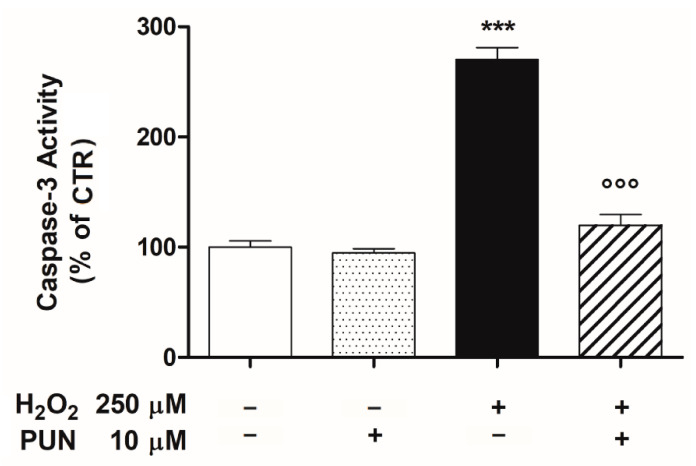
Pre–treatment with 10µM punicalagin (PUN) for 24 h downregulated the H_2_O_2_–induced caspase–3 activity in ARPE–19 cells. Data are expressed as a percentage relative to the untreated cells (control = 100%) and are presented as the means ± SEM of 6 replicates per group from two independent experiments. One–way ANOVA analysis was carried out followed by post hoc Newman–Keuls test. *** *p* < 0.001 vs. Control; °°° *p* < 0.001 vs. H_2_O_2_ alone.

Oxidative damage linked to the permeabilization of the mitochondrial outer membrane causes the release of cytochrome c, which activates a family of proteases, named caspases. Among these, caspase–3 in particular amplifies the cell death signal. Consequently, we examined if PUN was able to modulate the H_2_O_2_–induced activity of caspase–3 in our experimental paradigm. A significant and consistent increase of caspase–3 activity was elicited by oxidative stimulus (250 μM H_2_O_2_) after 24 h of incubation in ARPE–19 cells. As shown in Figure 5, caspase–3 activity was increased by 2.7–fold in the H_2_O_2_–treated group compared to the untreated control group. Under these conditions, pre–treatment with 10 μM PUN for 24 h was able to inhibit the enzymatic activity of the caspase–3 stimulated by oxidative damage, bringing it back to the untreated control levels (Figure 5). At the same concentration (10 μM), PUN did not affect the basal enzymatic activity of caspase–3 (Figure 5).

## 4. Discussion

Both exogenous and endogenous oxidizing agents can cause oxidative stress in RPE, triggering the cascade of pathological events underlying the onset of numerous retinal neurodegenerative diseases, such as age–related macular degeneration, retinitis pigmentosa, and diabetic retinopathy [43]. The oxidative stress process is driven by an imbalance between pro–oxidant species and antioxidant defenses, which leads to a progressive alteration of the redox regulation signaling mechanisms associated with ROS production increases. A high production level of ROS leads to cellular dysfunctions mainly in the mitochondria, which lose their membrane integrity, with consequent dissipation of the mitochondrial membrane potential (ΔΨm) and malfunction of the respiratory complexes [44,45]. Therefore, the attempt to counteract the overproduction of ROS, which are considered the mediators of oxidative damage, is one of the most studied approaches for preserving RPE functional integrity upon oxidative stress [46]. Emerging research evidence suggests that natural antioxidant compounds, especially polyphenols and carotenoids, could reduce oxidative stress in RPE [47,48].

Punicalagin, a polyphenolic phytochemical responsible for more than the half of pomegranate juice’s antioxidant effects, possesses a wide range of biological activities in numerous different tissues and cell types [26,27,28,49]. Recently, our group demonstrated for the first time that PUN also has antioxidant properties in RPE, lowering exogenous oxidative–damage–induced ROS levels and improving cell viability via the Keap/Nrf2/ARE signaling pathway [33]. In the present study, we reinforce this hypothesis by extending the antioxidant action range of the PUN to the mitochondria of RPE cells.

The RPE cell oxidative stress model used in this study was obtained by treatment with 250 µM H_2_O_2_ for 24 h on the human–RPE cell line ARPE–19. This is a widely used model for in vitro studies of oxidative damage [34,50,51]. We report here that the H_2_O_2_ treatment markedly reduced the cellular viability and promoted mitochondrial dysfunction due to an increase of ROS mitochondrial levels and a reduction of mitochondrial membrane potential. However, these pathological changes were antagonized by pre–treatment for 24 with 10 μM PUN before the oxidative damage by H_2_O_2_, confirming the protective properties of the compound in RPE cells [33]. In particular, we found that PUN decreased H_2_O_2_–induced ROS generation and improved mitochondrial function, completely reversing mitochondrial membrane potential loss caused by the oxidative stimulus. This suggests that PUN can possibly restore mitochondrial respiratory chain activity in RPE cells.

The maintenance of mitochondrial viability depends on the ability of the mitochondria to detect changes in the redox state and respond commensurately to metabolic requests. During oxidative phosphorylation, electrons flow from the reduced state to the oxidized state along the electron transport chain, which is embedded in the inner mitochondrial membrane. The energy released by the flow of electrons (through complexes I, III, and IV) is used to pump hydrogen ions from the mitochondrial matrix to the intermembrane space, creating a concentration gradient that is positive on the outside and negative on the inside. [52,53]. If cellular respiration is slow or stops, the gradient created by cellular respiration fails, the produced energy decreases, and finally the cells die [54]. Our results clearly showed that PUN preserves the functionality of complexes I, III, and IV, counteracting their activity decline induced by H_2_O_2_. It is intriguing to note that both H_2_O_2_ and PUN had no action on complex II, probably due to its location on the inner mitochondrial membrane, which makes the complex less susceptible to oxidative stress [55]. Interestingly, complexes I, III, and IV have a proton–pumping function. When their activities decrease, the proton pump function is reduced, triggering a decline of mitochondrial membrane potential [56]. In particular, the inhibition of enzymes of complexes I and III increases the production of O_2_^−^, causing an increase in oxidative stress. In addition, the reduction of the activity of complex IV, which plays a key role in the regulation of aerobic energy production, can result in compromised membrane potential and a reduced ATP level, and therefore severe mitochondrial dysfunction.

The oxidative damage linked to the alteration of the outer mitochondrial membrane permeabilization causes the release of cytochrome c in the cytosol, which promotes the activation of caspases. In particular, cytochrome c induces activation of downstream caspase–3, which triggers apoptosis and processes cellular death. In addition, caspase–3 itself favors further cytochrome c release from mitochondria, which amplifies the death signal [57,58]. Consequently, we examined in our experimental paradigm PUN’s effects on caspase–3 activity in RPE cells exposed to H_2_O_2_. Exposure of RPE cells to H_2_O_2_ resulted in a sharp increase of caspase–3 activity, which was antagonized in a significant manner by pre–treatment with PUN 24 h before the addition of H_2_O_2_. Therefore, these results lead us also to hypothesize that PUN’s protective role against apoptosis is induced by the caspase cascade, triggered in turn by deterioration of mitochondrial functions.

The results of the present study on the one hand confirm and reinforce the observations of our previous study [33], while on the other hand provide important new information on the protective role of PUN against oxidative damage in RPE. We hypothesize that the protective mechanism underlying the effect of PUN in RPE has a dual pathway. In fact, it both stimulates cellular antioxidant systems through the Nrf2/HO–1 signaling pathway [33] and protects mitochondria via Nrf2 from oxidative damage by antagonizing mitochondrial dysfunction, which we evaluated in terms of mitochondrial membrane polarization loss and through mitochondrial respiratory chain complex inefficiency. Interestingly, our findings agree with current knowledge about PUN and its antioxidative action via Nrf2 pathways observed in other experimental models in vivo and in vitro [59,60]. Furthermore, recent studies have shown that Nrf2 also affects many aspects of mitochondrial physiology, such as the structural integrity and dynamics of this essential organelle [41,42]. However, exogenous H_2_O_2_ binds Keap1 in the cytoplasm, preventing binding and activation of Nrf2 [61]. In this contest, our study shows for the first time that PUN and the Keap/ Nrf2/ARE signaling pathway act in concert to maintain redox homeostasis, protecting the RPE cells from H_2_O_2_–induced oxidative stress [33]. Thus, it can be concluded that PUN has a critical role in maintaining mitochondrial integrity and redox homeostasis under conditions of oxidation. Obviously, further investigations in vitro will be needed to confirm the basic hypothesis of our studies, possibly on other cellular experimental models of RPE that are closer to the natural physiological model than the ARPE–19 model [62,63,64,65].

## 5. Conclusions

To the best of our knowledge, our study provides the first evidence that in RPE cells, PUN is able to counter the H_2_O_2_–induced, mitochondrial–dysfunction–associated pathological changes, such as the accumulation of ROS, the membrane potential reduction in mitochondria, and the increase of caspase–3 activity. Therefore, although some questions remain open, it is possible to speculate that mitochondria are targets for PUN antioxidant action in H_2_O_2_–treated RPE cells.

The results of our study add new insights into the molecular mechanisms underlying the antioxidant properties of PUN on RPE exposed to oxidative stress. We hypothesize a synergistic effect of PUN capable of maintaining both redox homeostasis and mitochondrial integrity and functionality, in turn avoiding the caspase cascade and consequent cell death.

Taken together, our findings indicate PUN as an effective nutraceutical agent, supplementation with which could represent a promising therapeutic approach for prophylaxis or treatment of oxidation–associated RPE disorders.

## Data Availability

Data are contained within the article.
